# Impact Analysis of 20-Week Multimodal Progressive Functional–Proprioceptive Training among Sedentary Workers Affected by Non-Specific Low-Back Pain: An Interventional Cohort Study

**DOI:** 10.3390/ijerph182010592

**Published:** 2021-10-10

**Authors:** Éva Anett Csuhai, Attila Csaba Nagy, Gergő József Szőllősi, Ilona Veres-Balajti

**Affiliations:** 1 Department of Physiotherapy, Faculty of Public Health, University of Debrecen, 26 Kassai Str., 4028 Debrecen, Hungary; balajti.ilona@sph.unideb.hu; 2 Department of Interventional Epidemiology, Faculty of Public Health, University of Debrecen, 26 Kassai Str., 4028 Debrecen, Hungary; nagy.attila@sph.unideb.hu (A.C.N.); szollosi.gergo@sph.unideb.hu (G.J.S.)

**Keywords:** workplace training program, low back pain, Multimodal Progressive Functional–Proprioceptive Training, occupational health, health promotion, Spinal Mouse, musculoskeletal diseases, musculoskeletal disease prevention

## Abstract

According to the latest data published by the WHO, 1.71 billion people suffer from musculoskeletal disorders and 568 million are affected by back pain, making these the most significant occupational health problems. The aim of this study was to analyze the effects of a newly developed Multimodal Workplace Training Program implemented among young sedentary employees in order to treat and prevent these problems. The 20-week Training Program was conducted at the National Instruments Corporations’ Hungarian subsidiary in Debrecen between January and June, 2019. Pre- and post-intervention questionnaires were used to assess subjective parameters. Baseline and follow-up physical examinations were performed using the SpinalMouse, Y-Balance, Sit and Reach, Prone and Side Plank, Timed Abdominal Curl, and Biering-Sorensen tests. The results for 76 subjects were eligible for statistical analysis. Our Training Program was effective in several aspects, including a reduction in musculoskeletal symptoms and improvements in posture (*p* < 0.001), in dynamic (*p* < 0.01) and static-isometric (*p* < 0.001) core strength, in flexibility (*p* < 0.001), in spinal inclination in the sagittal (*p* < 0.001) and frontal (*p* < 0.01) plane, and in balance and coordination (*p* < 0.05). The Multimodal Progressive Functional–Proprioceptive Training was highly effective, and the application of such a complex training program can be recommended in workplace settings.

## 1. Introduction

According to a review of the latest results of the Global Burden of Disease Study 2017 (GBDS) published by the World Health Organization (WHO), musculoskeletal diseases (MSDs) and, especially, low back pain (LBP) are a major public health problem around the world. Compared to the data collected in 1990, in 2017, out of 354 diseases and injuries, LBP still remained the leading cause of the total number of years lived with disability (YLDs) in 126 of 195 countries and territories [1]. The increase in global YLDs accounted for by LBP from 1990 (42.5 million) to 2017 (64.9 million) was 52.7%, and the highest number of YLDs was found in Western Europe [2]. Around 1.71 billion people (95% UI: 1.63–1.80 billion) suffer from MSDs, and 568 million (95% UI: 505–641 million) are affected by LBP, resulting in 64 million (95% UI: 45–85 million) YLDs globally. Moreover, back pain is the leading cause of work absenteeism, loss of productivity and premature exit from the workforce among the adult population, causing an enormous direct and indirect financial burden for the health-care system, the employer and the employee [3,4].

Reports made by the European Agency for Safety and Health at Work (EU-OSHA) and the National Institute for Occupational Safety and Health (NIOSH—USA) refer to prolonged sitting and awkward static postures as some of the major risk factors for the development of LBP as well as other musculoskeletal, cardiovascular and metabolic disorders. The intention of these organizations is to reduce the negative health impact of sedentary work by collecting data on risks and symptoms, prevalence and incidence, work absenteeism, and health-related expenditures, and help employers by creating guidelines and sharing best practices in workplace settings. The reduction of sedentary time, shifting to standing and walking meetings, using stairs instead of elevators, the application of sit–stand workstations, flexible rest breaks, ergonomics education and the allowance of time for physical activity are among the most recommended methods for the prevention of LBP [5,6,7].

Based on a meta-analysis published by Sowah et al. [8] in 2018, an occupational physical exercise program alone or besides additional theoretical education was found to be effective for the prevention of LBP. Education itself (e.g., back school, instruction and training on ergonomics and proper manual handling, videos and pamphlets), the application of foot orthoses, shoe insoles and passive lumbar supports (e.g., braces and belts, chair back rests, etc.) were found to be ineffective. Insufficient evidence was found in relation to the modification of workplaces or working stations; therefore, no clear conclusions can be drawn in this aspect [8]. The findings of the meta-analysis of Moreira-Silva et al. [9] supplied evidence of the effectiveness of workplace physical activity interventions on general musculoskeletal pain; however, only low-quality evidence suggested the effectiveness of such interventions for LBP. The authors concluded that further studies were required due to the scarcity of literature [9].

In order to address the problem, it is important to primarily classify LBP into two subgroups of specific and non-specific LBP, to create the best preventive or therapeutic regimen. Low back pain is a symptom that can be caused by several different sources. Close to 90% of all cases have non-specific sources, which means that the patho-anatomic cause of the symptom cannot be identified using any reliable examination device or imaging. Possible contributing factors may include a poor posture, muscular disbalance, the overuse of spinal musculature, mental stress, vibration, rotation with lifting weight, long term static sitting or standing, a low level of physical activity, overweight, etc. All the remaining cases are in the class of specific LBP, which is caused by radiculopathy and radicular pain, spondyloarthropathy, compression fractures, epidural abscesses, cauda equina syndrome, spinal canal stenoses, malignancy or referred pain due to other internal organ diseases, etc. [10].

The effects of recurrent LBP on the function of the lumbar musculature may be detrimental in the long run. The co-contraction of the multifidus, transverse abdominis, pelvic floor and diaphragm muscles increases intra-abdominal pressure, therefore creating a stable core and stabilizing the spine on the intervertebral level, which is highly required to manage internal and external forces acting on the inter-vertebral joints. These muscles provide segmental core stability, while the superficial or global muscles are mainly responsible for dynamic positioning, the mobility of the trunk and creating its general stabilization. The density of muscle spindles in the deeper layer of multifidus muscles and in fascicles closer to the facet joints is larger, which plays an important role in proprioception, hence acting as “kinesiologic monitors”. Unfortunately, multifidus muscles can be inhibited by the motoneuron reflex due to low back pain or any other arthrogenic source, which can lead to the overloading of superficial muscles, recurrent pain, segmental instability and other intersegmental complications such as disc protrusion, prolapse or herniation [11,12].

Three dominant signs of degeneration due to the inhibition of the multifidus can be detected by imaging: a decreased radiographic density, a decreased cross-sectional size (atrophy) and fatty infiltration. Fatty infiltration is highly related to LBP, and MR spectroscopy has confirmed that the fat content of the multifidus is significantly higher in LBP subjects compared to asymptomatic controls [13]. According to the findings of Hides et al. [14], the spontaneous recovery of the function of the multifidus cannot be expected after the remission of symptoms. In contrast to those who were exercised, those subjects who did not receive specific exercises recovered in all the other examined aspects, but the atrophy of the multifidus was still seen by 10 weeks after “complete” recovery [14].

In order to prevent these adverse consequences, it is important to pay special attention to the activation of the multifidus and core muscles. However, a number of other aspects also need to be considered, such as improving posture, improving muscle flexibility or ergonomic education as part of multidimensional training. Several previous studies have focused on the effects of various types of exercise interventions to prevent or treat LBP. According to the systematic review and meta-analysis by Gomes-Neto et al. [15], segmental stabilization (SS) exercises were as effective as manual therapy and more effective for pain intensity and disability than general exercises of the superficial muscles. However, the authors emphasize that the data used in the analysis showed significant heterogeneity in terms of the exercise program duration, progression criteria, type of muscle activation, and feedback methods; thus, further randomized controlled trials are required [15]. A randomized controlled trial was conducted by Sipavicienea and Klizieneb [16] for the comparison of the effects of the lumbar stabilization (*n* = 35) and the lumbar muscle strengthening (*n* = 35) exercise programs among female sedentary workers suffering from LBP. The length of both interventions was 20 weeks, and training sessions were performed twice a week for a time period of 45 min. The results demonstrated that both programs were effective in the reduction of LBP symptoms and functional disability and in an increase in the cross-sectional size of the multifidus, although lumbar stabilization proved to be more effective [16]. A systematic review with a meta-analysis published by Tong et al. concluded that impairment of lumbar proprioception can be detected among subjects affected by LBP [17]; thus, the application of any proprioceptive task possibly improves the function of the lumbar musculature. Areeudomwong et al. [18] used the Proprioceptive Neuromuscular Facilitation (PNF) technique to address LBP symptoms and balancing abilities in a case–control study. The intervention performed by a physiotherapist lasted for only three weeks, with only 30 to 40 min sessions organized three times a week. The progression of the exercises was determined by the weekly changing tasks. Although the training was short and the number of subjects was small, the results showed a small but positive effect on pain intensity, disability and static balancing ability [18].

The facts mentioned above support the urgent need for adequate prevention and effective treatment methods for the management of risks and symptoms by targeting the largest number of subjects at risk or affected by the problem to create a significant impact in as short a period of time as possible.

The aim of our study was to perform the impact analysis of a newly developed Multimodal Workplace Training Program among sedentary workers by examining physical parameters (posture, spinal mobility, muscular flexibility, static and dynamic muscle strength, coordination and balancing abilities) and subjective outcome measures.

## 2. Materials and Methods

**Figure 1:**
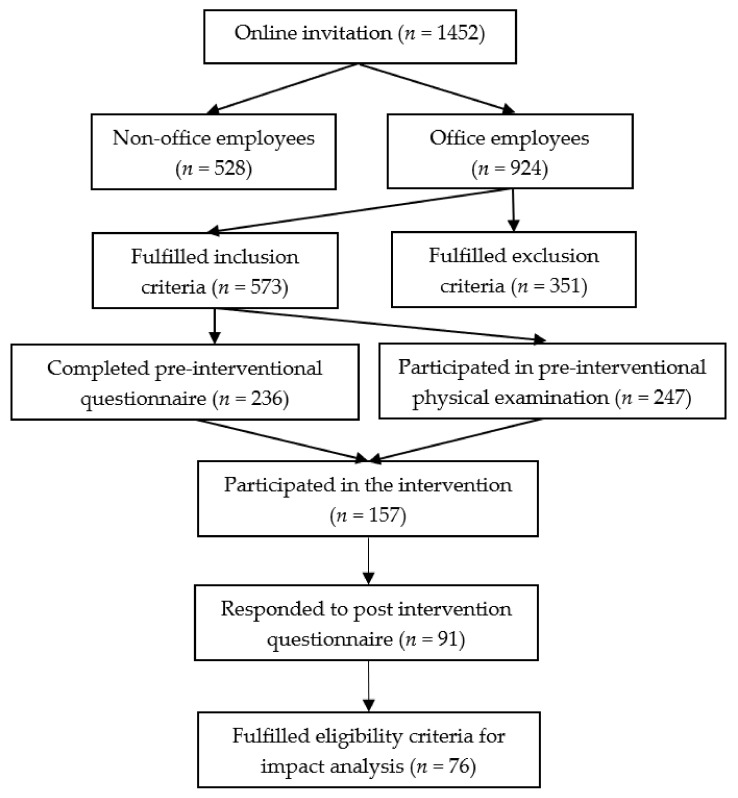
Flow diagram of subject selection for the study.

An interventional cohort study was conducted among sedentary employees of the Hungarian subsidiary of the National Instruments Corporation (Austin, Texas, USA) in Debrecen at NI Hungary Ltd. Pre-intervention examinations were performed between the 9th of November and the 7th of December 2018. The intervention was implemented in the company’s fitness center between the 28th of January and 14th of June 2019. Post-intervention tests were conducted between the 17th and 28th of June. From a total of 1452 employees, those workers who had experienced lumbar complaints in the previous year and who had sedentary occupations were invited to participate in the physical examinations and in a 20-week long Multimodal Progressive Functional–Proprioceptive Training Program specifically designed for employees in the workplace environment during their working hours. An online screening questionnaire for the collection of subjects and a motivation letter about the schedule and content of the physical examination and the “New Spine Program” were distributed via the electronic newsletter of the company in October. The invitation contained the inclusion and exclusion criteria (Table 1); information about the dates, venues and process for the physical examination; the content and schedule of the Training Program; and the method of registration for both the physiotherapeutic assessment and training sessions. Employees were asked to complete a pre-interventional questionnaire if they fulfilled the inclusion criteria and were willing and able to participate in both the physical examinations and the intervention itself (Figure 1).

During the Week of Recognition—a series of programs for workers at the company—held in October, two introductory presentations about the “New Spine Program” were given by the leading physiotherapist of the study. The completion of the self-administered questionnaire and attendance at the physical examination were prerequisites for participation in the Training Program, although the completion of this questionnaire was not a prerequisite for participating in the pre-interventional examinations as requested by the employer.

The pre-intervention self-administered questionnaire (preIQ) was distributed among the entire staff of the company; the pre-intervention physical examination (preIPE) was designed for a limited number of 250 applicants; the Training Program (TP) was organized for a maximum of 160 participants; the post-intervention self-administered questionnaire (postIQ) was sent to those who participated in the TP, and the post-intervention physical examination (postIPE) was organized for those who fulfilled the eligibility criteria.

The baseline physical examinations were organized and performed in the company’s medical room and fitness center with the assistance of 18 physiotherapy students. The assistants were practiced in the given examination method and performed the same examination during the pre- and post-examinations. The physical examination of one subject took an hour to complete and took place with a rotating system in ten different examinations. The order of the tests was different for each subject who started the testing procedure at the same time, as a “rotating system” was used in order to decrease the required time for the physical examination. The employer stipulated that the employees should complete the whole process at least within an hour; therefore, we used different, predefined, printed timetables to help the employees to follow their own order of tests to prevent congestion and to speed up the process. The time length for recovery between the tests was determined as a minimum of 2 up to a maximum of 5 min, taking into account subjective exhaustion using the Borg-scale—Rate of Perceived Exertion (RPE). The subjects were allowed to perform the next physical test only if their RPE was a maximum of 1–2 on the Borg CR 10 Scale [19]. The recovery time was extended in those rare cases when the subject failed to recover in 5 min. The length of the rest interval was determined according to the recommendation of the American Society of Exercise Physiologists on the accurate assessment of muscular strength and power in order to prevent interference in the results that could have been caused by the random conduction of the tests with inappropriate rest intervals [20,21,22]. The preIPE took five and the postPE took two days to complete. The participants were asked to generate their own study code using specific numbers extracted from their national ID numbers, the initials of their first names and last names, and their mothers’ years of birth. These study codes were used during data collection in the physical examination, in the registration for the training sessions, in signing the attendance lists and also as passwords for the downloading of the preIPE and postIPE result-sheets from an online interface.

The intervention was conducted by a physiotherapist at the fitness center of the company on every workday alternately in the morning and afternoon. Four training sessions were held daily for a time period of 30 min each. A maximum of 20 participants were allowed to register online for each session, and were able to choose the date and time in the timetable, according to their daily job responsibilities and workload. The adherence rate was measured using an attendance list, and signatures were collected in relation to the study codes. Besides physical exercises, every session contained a theoretical explanation of proper posture, the correct performance of exercises and functional tasks, the ergonomic characteristics of work while sitting, standing and carrying weight, etc. The progression of the program was determined weekly by a training schedule, and according to this, all the sessions contained exactly the same exercises, number of repetitions and theoretical content. The subjects were asked to attend the trainings three times a week if possible. The minimum requirement for the statistical analysis of the results of the preIPE and postIPE was attendance at a minimum of 50% of the recommended 60 sessions.

All the attendants were asked to complete the postIQ at the end of the program. Those participants who attended a minimum of 30 sessions or more were invited to participate in the postIPE.

### 2.1. Pre-Intervention Questionnaire

The online survey contained questions on gender, age, perceived health, musculoskeletal symptoms in the last year, painful regions, the time spent sitting on workdays and weekends, and the intention to participate in the physical examination and in the Training Program led by a physiotherapist. Questions on the level of daily physical activity were also applied based on the International Physical Activity Questionnaires (IPAQ), but these results will be published in the future.

### 2.2. Physical Examination

#### 2.2.1. Spinal Mouse

The overall and regional spinal mobility and posture were measured in the sagittal and frontal planes both in sitting and standing positions using SpinalMouse (Idiag, Volkerswill, Switzerland), a wireless electronic measurement device [23]. The preparation and the sagittal plane examination were performed according to the same procedure explained in the previous publication of our pilot study [24].

#### 2.2.2. Frontal Plane, Standing Position

1. Neutral in standing: The subject was asked to maintain a relaxed position with the feet shoulder width apart, with straight knees and arms by their side, looking and facing straight horizontally forward.
2. Maximal left and right lateral flexion in standing: The subject was asked to cross their arms in front of their chest and put their hands on their contralateral shoulder, and then move their trunk to the left with straight knees in a full range of motion (ROM) with slow movement from segment to segment, while keeping their trunk in the frontal plane, without any rotation. The examination was performed for the right side as well.

#### 2.2.3. Frontal Plane, Sitting Position

The examination was performed on a height adjustable chair in order to secure 90 degrees in the ankle and knee joints.

1. Neutral in sitting: The subject was asked to maintain a relaxed sitting position with the feet shoulder width apart, arms by their side, looking and facing straight horizontally forward.
2. Maximal left and right lateral flexion in sitting: The subject was asked to cross their arms in front of their chest and put their hands on their contralateral shoulder, and then move their trunk to the left in a full ROM with slow movement from segment to segment, while keeping their trunk in the frontal plane, without any rotation. The examination was performed for the right side as well.

The examination was performed only once with no warm-up or practice before, according to the verbal instructions of the examiner [24,25].

The overall and regional posture and inclination of the spine were determined in each position and plane, compared to a vertical axis perpendicular to the ground. Based on the raw data collected by the measurement device, the relative positions of the vertebrae and the vertebral column were used by the software, and the total and regional ROMs of the spine were estimated. Raw and estimated data from the measurements are presented by the software in a table according to the following: the values from upright (U), flexion (F) and extension (E) in the sagittal plane, where the kyphosis angles are positive, and the lordosis angles are negative values; the values from upright (U), left lateral flexion (LE) and right lateral flexion (RI) in the frontal plane, where the values for the left direction compared to the vertical axis are positive and the values for the right direction compared to the vertical axis are negative. The estimated ROM values are the following: the range of flexion from upright (U–F), range of extension from upright (U–E), total range from extension to flexion (E-F), range of left lateral flexion from upright (U–L), range of right lateral flexion from upright (U–R) and range from left to right lateral flexion (L–R). Only data related to the total inclination and segmental (thoracic-, lumbar- and sacral spine/hip) mobility of the spine were used for the statistical analysis.

#### 2.2.4. Chest Mobility

Chest expansion was measured at three levels of the thorax using a measuring tape. Measurements were taken during complete inspiration and complete expiration at the level of the axilla (CEA), the level of the xiphoid process of the sternum (CEX) and the level of the tenth ribs (CER). The chest expansion on each level was determined by subtracting the expiration values from inspiration values. The examination was performed only once following 2 or 3 repetitions of forced inhalation and exhalation according to the verbal instruction of the examiner [26,27].

#### 2.2.5. Sit and Reach Test

The Sit and Reach test for the examination of spinal and hamstring flexibility was performed using a Sit and Reach Box (SRB) (Cartwright Fitness Ltd., Chester, UK). The SRB was set to the baseline of 15 cm. The participants were asked to sit on the floor in a long-sit position, with full extension of the knees, while the soles of the feet were positioned against the surface of the box with a neutral position for the ankle. After two repetitions of warm-up practice, two measurements were recorded by the examiner. A ruler was used to mark the “zero” point, from which it was pushed forward on the superior surface of the SRB. The subjects were asked to place the dominant palm on the contralateral one facing downward and bend forward with the trunk as far as possible, with the knees kept extended and sliding the ruler on the SRB with the tip of the fingers. Statistical analysis was performed in relation to both measurements and the average of those as well [28].

#### 2.2.6. Y-Balance Test

The instrumented version of the lower quarter Y-Balance Test (YBT) (Functional Movement Ltd., London, UK) was primarily used to determine the dynamic balance of the lower extremities. The participants were asked to stand on the center of the equipment with one leg and reach with the contralateral leg toward the anterior, posteromedial and posterolateral directions, while pushing a slider on the bars located on those directions as far as possible. All three bars present the reach distance in cm; hence, the results can easily be registered. The subjects were asked to practice the task three times on each side in each direction prior to the three recorded trials. They were also asked to maintain a single leg squat stance, while keeping their hands on their hips and full contact of their foot on the supporting surface, without touching the ground with their contralateral foot. If the trial failed to meet these requirements, the result was unsuccessful. Practice and recorded trials started with a right foot stance on the YBT platform, and after the completion of three trials for one reach direction, the subjects were allowed to perform the same reach direction with a left foot stance. The results of the trials were averaged and recorded in each direction without leg length normalization [29].

#### 2.2.7. Plank Test

The Prone Bridging Test was used to measure the static, isometric core strength. The participants were directed by one tester via verbal and manual assistance, and were asked to get into a prone forearm plank position and hold it correctly as far as possible: both forearms were placed on the floor, parallel to the midline; the palms were facing the floor, and the elbows were placed under the glenohumeral joints; both humeri were perpendicular to the ground; both scapulae were stabilized on the plane of the dorsal thorax; the head, neck, trunk and lumbo-pelvic–hip complex were in a neutral position; the knees were extended; the toes were placed on the floor, and the ankles were in a neutral position. Another tester inspected the subject and started a digital timer when the correct posture was located. The timer was stopped as soon as voluntary termination of the test occurred due to any reason; the tester noticed or the participant reported signs and symptoms of exhaustion; or the subject repeatedly failed to maintain the proper position—after a first verbal warning from the investigator. The test was conducted only once according to the verbal and visual instructions of the examiner, without warm-up or practice, and the results were recorded in seconds [30].

#### 2.2.8. Side Plank Test

The Side Bridging Test was used to measure the static, isometric strength of the left- and right-sided core musculature. The participants were asked to suspend the whole torso, keeping its neutral position from a starting side-lying position on a mat, while only the edge of the foot, the elbow, the forearm and the hand were supported by the floor on the test side, and they were asked to maintain the side bridging as far as possible. The side plank was performed on both sides, with a neutral lumbo-pelvic–hip complex, in line with the trunk, neck and head, with the extension of the knees and a neutral ankle position on both sides. The superior ankle was placed on the inferior one, and the superior arm was beside the trunk. The supporting shoulder was abducted to circa 80–85° without rotation; the elbow was placed just beneath the glenohumeral joint, so the humerus was perpendicular to the ground, and the forearm was supported in a pro-supinated position. The same testing process was used as described above in the Plank Test; the procedure was performed with right-side prior to left-side bridging [31,32].

#### 2.2.9. Timed Abdominal Curl Test

The Timed Abdominal Curl Test was used to measure the dynamic endurance of the superficial trunk flexor muscles. The subjects were asked to lie down on a mat in a supine position while the lower limbs were supported by a height-adjustable box at 90–90° of flexion in the hip and knees and with arms crossed in front of the chest. The participants were instructed to lift up the head, neck and shoulder until the elbows were touching the thigh while the lumbar spine and pelvis were stabilized on the ground. The pace of the performance was given by the sound of a metronome (50 beat/min), and the cadence was set to 25 repetitions/min; thus, equal time was spent for curling up and down. The recording of the time and repetition number was started at the beginning of the exercise and ended immediately at the end of the last correct performance. The test was conducted only once according to the verbal and visual instructions of the examiner without warm-up or practice, and the results were recorded in seconds and as the repetition number [33,34].

#### 2.2.10. Modified Biering-Sorensen Test

The static, isometric trunk extensor endurance test was performed according to the modified version of the method of Biering-Sorensen et al. [35] The participants were positioned on the examination table in a prone position with the lower limbs and the pelvis supported. The trunk was unsupported by the table, but a chair was used to help the subject in manually carrying the trunk while the legs and pelvis were stabilized by three mobilization belts with metal buckles. The subjects were asked to place the elbows laterally, put their hands under the forehead and lift up the trunk to the horizontal plane. As soon as a horizontal position was reached, an inclinometer was placed on the back of the trunk, between the scapulae. At this moment, a digital timer was started, and when the subject failed to further maintain the position, the test was terminated. The test was conducted only once according to the verbal instructions of the examiner, without warm-up or practice, and the results were recorded in seconds [35].

#### 2.2.11. Dynamic Trunk Extensor Endurance Test

The test was performed, but the results were discarded due to measurement bias, caused by the replacement of two raters of the post-intervention examination in this one case.

### 2.3. Multimodal Progressive Functional–Proprioceptive Training Program

Our “Multimodal Progressive Functional–Proprioceptive Training Program” is a newly developed and unique physical exercise program, which was specifically designed for workplaces by addressing the special needs and characteristics of sedentary employees in order to promote their health and to reduce occupational musculoskeletal diseases and non-specific LBP. The Training Program (TP) was structured by a weekly changing progressive schedule (Table 2) The foundation of the training was the segmental stabilization in order to activate and re-educate the main deep superficial stabilizers of the lumbar spine in a precisely positioned neutral–anatomical position. The exercises built on this basis progressed from a lying through sitting to standing position, from a stable and larger to an unstable and smaller support surface, and from simple to complex tasks; thus, more complicated exercises with multiplanar movements were progressively performed [16,36]. The training was multimodal, since ergonomic education, stretching, self-myofascial release, segmental stabilization, the isometric and isotonic strengthening of the muscular system, the stimulation of the proprioceptive system and dynamic functional training exercises were applied.

Every training session started with 5 min mobility and light aerobic exercises. Except for the stretching sessions—which started with a 10 min warm up—every training session ended with a 5 min stretching exercise preferably for the main muscles involved. The performance of the exercises and segmental stabilization were inspected; an inappropriate position was corrected verbally and/or manually if required. The progression of the exercises was determined by increasing the length of the exercise: (a) from the basic 3–5 s gradually up to 30 s during the 3rd–4th week; (b) from the basic 15 s gradually up to 30 s during the 5th–6th week; (c) from the basic 30 s up to 45 s during the 7th–8th week. Breathing exercises were performed during rest periods of (a) 3–5 s, (b) 5–10 s (c) and 10–15 s. The exercises were performed in 4 repetition sets with longer rest periods in between. The numbers of sets during the sessions were dependent on the given length of each exercise and rest periods. From the 9th week up to the 20th week, the length of an exercise was 45 s with a 15 s rest period in 4 repetition sets, and 4 sets of different exercises were performed during a session. The participants were allowed to have some rest during the exercises according to their subjective sensation of fatigue.

#### 2.3.1. Ergonomic Instructions, Stretching Techniques and Self-Myofascial Release

**Figure 2:**
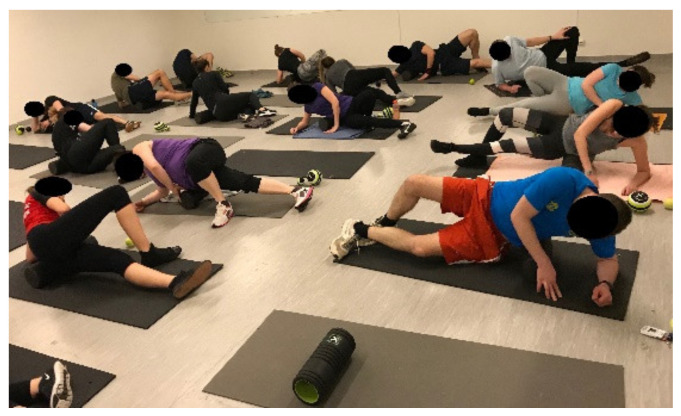
Self-myofascial release.

This phase was intended to teach and enable participants to practice the active and passive stretching exercises used during the TP, first without and then with tools (e.g., a towel). Pain relief, myofascial compression and self-massage were performed using a self-myofascial release (SMR) foam roller (All-Right Bt. Budapest, Hungary) and TriggerPoint MB5 (12.7 cm) foam massage balls (Trigger Point Performance Inc., Austin, TX, USA). The theoretical education was intended to teach and to enable practicing ergonomic aspects related to work and physical activity (Figure 2).

#### 2.3.2. Segmental Stabilization of Lumbar Spine

**Figure 3:**
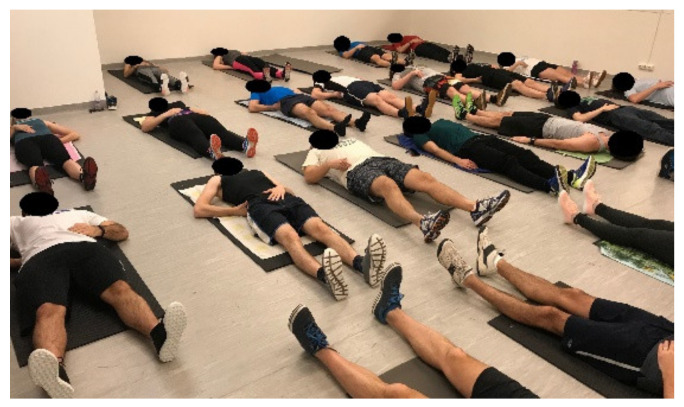
Segmental stabilization.

The goal of this phase was to teach and enable the practice of conscious, voluntary segmental lumbopelvic stabilization using the abdominal drawing-in maneuver (ADIM) [37]. The participants were asked to lie on the mat in a straight but relaxed position and search and feel for the proper neutral position of the pelvis and lumbar spine. Verbal explanations helped in the required positioning, and the subjects had been taught to manually palpate and control the anterior superior iliac spine (SIAS) and symphysis pubis in line with the horizontal plane. After correct positioning, the subject was taught—following Saliba et al. [38]—to contract the deep core musculature, focusing on the multifidus, abdominal transverse, and pelvic floor muscles, without the contraction of superficial muscles or any movement of the pelvis and spine. The verification of proper contraction was carried out by both the physiotherapist and the subject. Supine bridging was performed with a slow hip extension by raising the pelvis and the trunk in a block by hip extension until reaching the trunk and femur in line (Figure 3).

#### 2.3.3. Static Strengthening of Core Musculature

**Figure 4:**
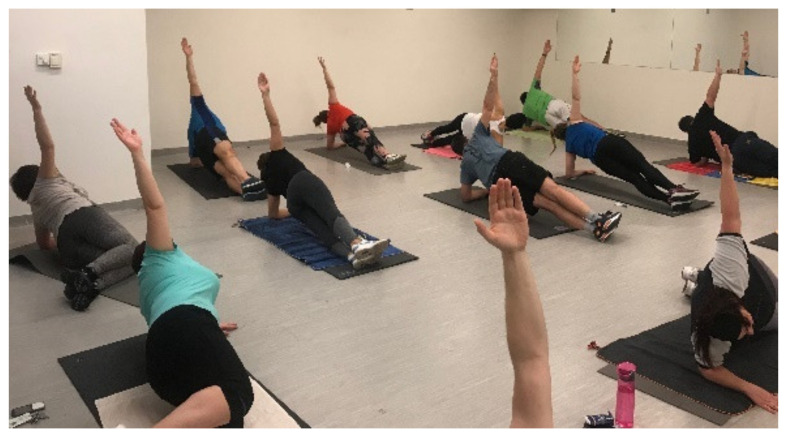
Segmental stabilization in side bridging.

**Figure 5:**
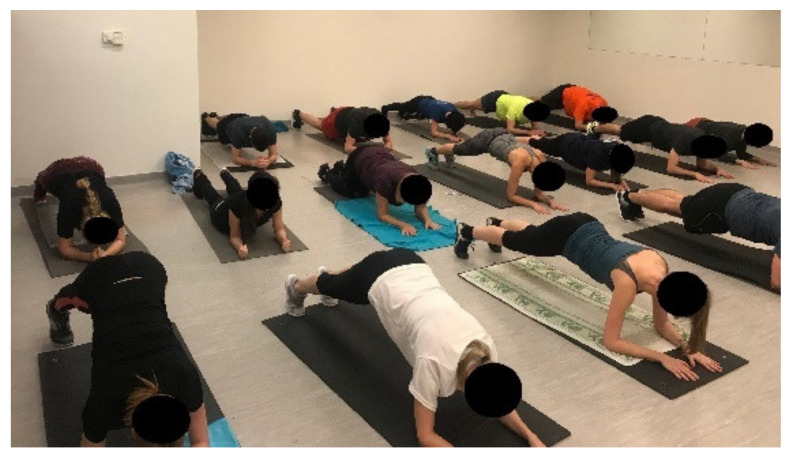
Prone bridging (plank).

The aim of this phase was to maintain segmental stabilization while performing static, isometric exercises: prone plank and side plank in a kneeling position. The position of the trunk and femur was the same as described above in the Plank and Side Plank Test, with the difference that the knees were bent at 90° and the superior arms were abducted toward the ceiling in side bridging. The position decreases the effect of gravity relative to the original method due to a shorter lever arm and increases stability due to a larger supporting surface. The original plank was performed by those who were able to progress during this week (Figure 4 and Figure 5).

#### 2.3.4. Segmental Stabilization Combined with Dynamic Exercises on Stable and Unstable Surfaces

**Figure 6:**
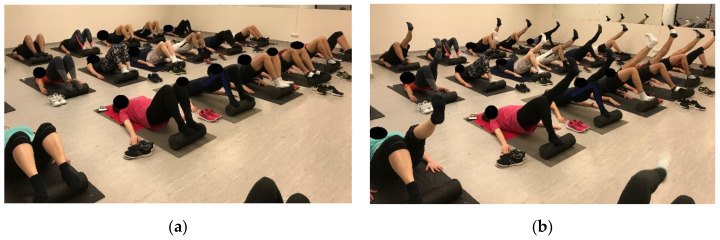
Segmental stabilization in unstable bilateral (**a**) and unilateral (**b**) supine bridging.

Focus was placed on an introduction to proprioceptive exercises by gradually decreasing the size of the supporting surface and switching from stable to unstable surfaces. In case of supine bridging, progression was determined according to the following: (1) bilateral stable bridging; (2) bilateral stable bridging with one-sided knee extension; (3) unilateral stable bridging; (4) bilateral unstable bridging; (5) bilateral unstable bridging with one-sided knee extension; (6) unilateral unstable bridging. Instability was created using a foam roller. An unstable supine plank was also used with bilateral prior to unilateral support. Instability was created using a Swiss ball (Figure 6a,b).

#### 2.3.5. Basic Functional Exercises in Different Positions

**Figure 7:**
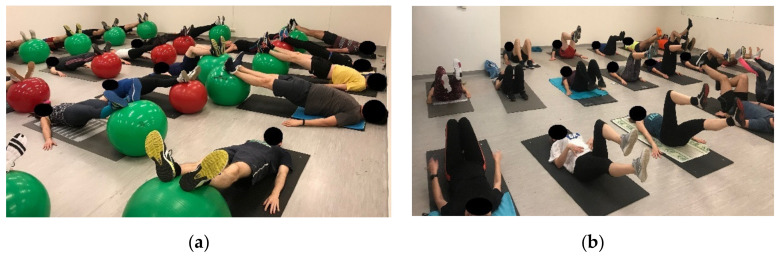
Segmental stabilization in unstable bilateral supine plank (**a**) and in stable unilateral leg lowering (**b**).

**Figure 8:**
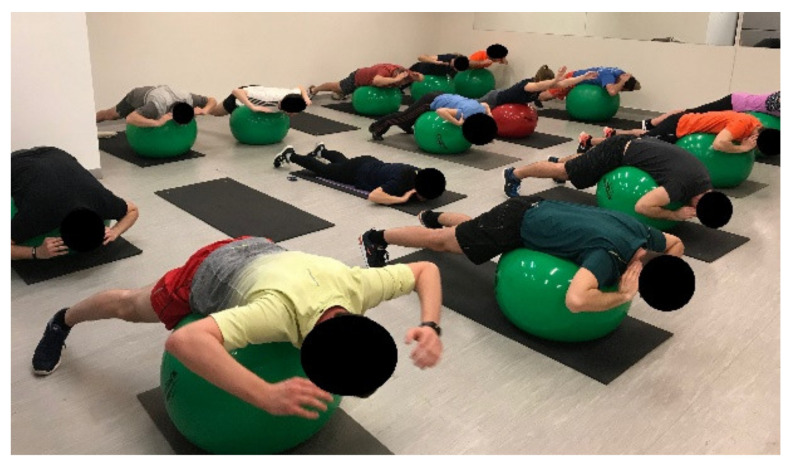
Unstable trunk extension.

A supine leg raise was performed besides core stabilization with one-sided lower limb support (hip–knee flexion, with feet flat); supine leg lowering was performed from a bilateral 90 degrees of hip and knee flexion to unilateral and then bilateral hip and knee extension (Figure 7b). A Swiss ball was used in sitting exercises for tilting the trunk to the anterior and posterior directions, in supine exercises for abdominal crunches and hamstring curls, and in prone exercises for back extension while maintaining segmental stabilization of the core. The standing exercises were lunges, squats and repeated bilateral side-step squats (Figure 7 and Figure 8).

#### 2.3.6. Functional Training

**Figure 9:**
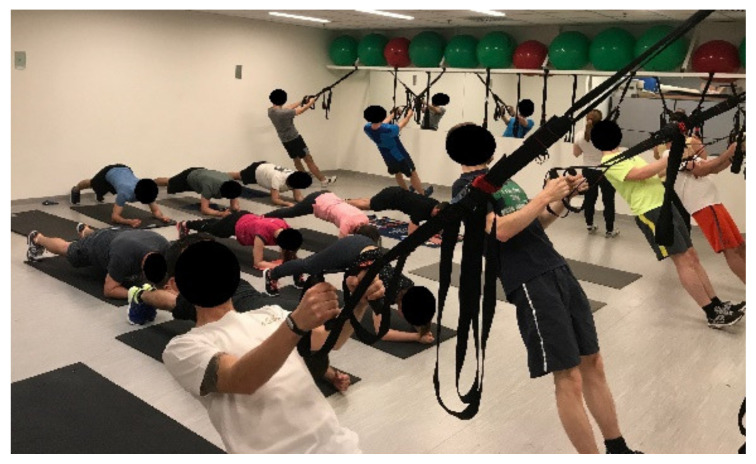
Functional training.

During the phase of Functional Training, the group was divided into two subgroups, and circuit training was performed. One subgroup performed exercises chosen from the previous phases of the TP; the other subgroup used a Suspension Trainer (ST) (TRX, Fitness Anywhere LLC, San Francisco, CA, USA) in order to perform unstable, multi-planar, functional exercises (Figure 9). As described above, in this section, 4 sets of different exercises were performed with 4 repetitions of each exercise. One repetition of an exercise lasted for 45 s, followed by 15 s of recovery, and a 1 min recovery time was allowed between the sets. None of the exercises were compulsory to complete; all the participants had the opportunity to ask for a given exercise to be changed to an easier or harder one. The exercises in this phase were chosen from those listed below, taking into account the principle of gradation, progression and individual subjective sensation of exhaustion or capability during the 12 weeks of functional training. Due to the factors listed above, the sequence and weekly progression of the exercises used in this phase cannot be generalized or defined precisely.

#### 2.3.7. Types and Progression of Functional Exercises Applied in the Phase of Functional Training

Squats

— Squat;
— Side-Squat;
— Swiss Ball Wall Squat;
— TRX-Squat;
— TRX Side-Squat;
— TRX Jumping Squat;
— TRX One-Leg Squat;
— TRX Inclined Squat;
— TRX Inclined Squat and Jump.

Lunges

— One-Sided Lunges;
— Alternating Lunges;
— TRX One-Leg Lunges;
— TRX One-Leg Lunges with Jump;
— Knee-Drive Jump.

Supine Bridging

— Bilateral Stable Bridging;
— Bilateral Stable Bridging with One-Sided Knee Extension;
— Unilateral Stable Bridging;
— Bilateral Unstable Bridging;
— Bilateral Unstable Bridging with One Sided Knee Extension;
— Unilateral Unstable Bridging;
— Swiss Ball Supine Bridging;
— Swiss Ball Supine Bridging with Bilateral Hamstring Curl;
— Swiss Ball Supine Bridging with Unilateral Support;
— Swiss Ball Supine Bridging with Unilateral Support and Hamstring Curl.

Types of Additional Functional Exercises

— Narrow and Wide Kneeling Push Up → Narrow and Wide Push Up;
— Abdominal Curl → Swiss Ball Abdominal Curl;
— Trunk Extension → Swiss Ball Trunk Extension;
— Kneeling Prone Plank → Prone Plank;
— Kneeling Side Plank → Side Plank;
— TRX Low Row;
— TRX Biceps Curl;
— TRX Y-fly Push Up;
— TRX Chest Fly;
— TRX Chest Press;
— TRX Supine and Prone Plank;
— TRX Supine and Prone Plank with Abduction of Lower Limbs;
— TRX Hamstring Curl.

#### 2.3.8. Suspension Trainer

The ST equipment contains an anchor and length-adjustable training straps with foot cradles and handles; hence, it can be used either to grasp with the upper extremities or to support the lower extremities. The STs were anchored vertically hanging 1.8 m off the ground, and the bottom of the foot cradles were approximately 8 cm from the ground. The amount of resistance of the “own body weight” was changed (increased or decreased) using different inclination angles for the body depending on the ability of the participant. The closer the center of gravity was located to the ground, the harder the exercise was. Proprioceptive stimuli were determined by the movable straps of the equipment and the small surface contact of the stance with the ground. All the exercises were performed with a focus on proper segmental stabilization.

Simple “pulling type” exercises (e.g., low row, biceps curl, Y-fly, etc.) require a heel stance while facing the anchor point of the equipment in an inclined position with a completely straight body. In order to maintain the anatomical position of the lumbopelvic complex, the alignment of the head and spine, a neutral position for the hips, the full extension of the knees and a neutral position for the ankles are required. A heel stance was required; touching the floor with the sole of the foot was disallowed since it may disrupt biomechanical alignment.

Complex “pulling type” exercises (e.g., a squat, lunge with row, one-leg squat, side squat, jump-squat, etc.) can be performed with full contact of the foot with the ground or with a calf raise.

Simple “pushing type” exercises (e.g., a push up, chest fly or chest press) require a toe stance, while facing the ground in an inclined position with a completely straight body. In order to maintain the anatomical position of the lumbopelvic complex, the alignment of the head and spine, a neutral position for the hips, the full extension of the knees and a neutral position for the ankles are required. A toe stance was required; touching the floor with the sole of the foot was disallowed since it may disrupt biomechanical alignment.

Complex “pushing type” exercises (e.g., an inclined squat, inclined squat and jump, and knee-drive jump) can be performed with full contact of the foot with the ground or with a calf raise.

Horizontal exercises were performed with feet placed into the foot cradles (e.g., a supine and prone plank, supine and prone plank with the abduction of the lower limbs, and hamstring curl).

### 2.4. Post-Intervention Questionnaire

The postIQ focused on the subjective opinions of the participants in relation to the TP. The questions among the others were focused on perceived health, on changes in previous symptoms and complaints, on changes in posture and endurance, on the adherence rate, on the perceived improvement of knowledge in health-related topics, on the musculoskeletal symptoms during the TP, on the usefulness of the content learned during the TP, on the capability of incorporating the content learned during the TP in daily living, and on the exercises that were practiced at home. The Net Promoter Score (NPS) was used to assess how much the participants supported and recommended the TP. The score divided the respondents into three subgroups of “detractors” (0–6), “passives” (7–8) and “promoters” (9–10) according to the points (0–10) given by the subjects. To calculate the NPS, we subtracted the percentage of “detractors” from the percentage of “promoters”. The classification of the TP related to the satisfaction and loyalty of the participants can be determined according to the NPS as follows: a need for improvement between −100 and 0%, a good rating between 0 and 30%, a great rating between 30 and 70%, and an excellent rating between 70 and 100% [39].

### 2.5. Sample Size Calculation

An a priori sample size calculation was performed with a power level of 90% and an α level of 0.05 using means (45.22 and 49.81) and standard deviations (7.21 and 6.99) relating to the flexibility of the spine and hamstring muscles based on the study of Muyor et al. [40].

### 2.6. Statistical Analysis

A Shapiro–Wilk test was used to check the normality of the continuous variables. Since most of the data did not follow normal distributions, non-parametric Wilcoxon signed-rank tests were used. Categorical data were analyzed using chi-square tests. Data are presented as medians and interquartile ranges (IQR) in the case of continuous variables and as percentages in the case of categorical variables. The Spearman rho-s were calculated in order to check the correlation between the variables related to the physical examination. The results were considered as significant if the *p*-values were below 0.05. The data were processed using Microsoft Excel and the Intercooled STATA version 13.0 software.

### 2.7. Ethical Approval

The study was approved by the Ethics Committee of the University of Debrecen (5103-2018), and the participants gave informed consent.

## 3. Results

An open call for the study was introduced to the employees of the company. After voluntary application, a total of 236 subjects completed the preIQ, 247 subjects participated in the preIPE, 91 subjects completed the postIQ, and 76 subjects were eligible for the postIPE, which is above our calculated minimal needed sample size (*n* = 51).

### 3.1. Pre-Intervention Questionnaire

For an open-call invitation, a total of 236 persons completed the preIQ, 109 male and 127 female participants with a mean age of 32.94 ± 7.26 years. Although 99% of the respondents thought that they could do a great deal for their own health, the perceived health of the respondents was “Good” for only 66% and “Satisfactory” for 32%. Answers relating to the region of musculoskeletal symptoms showed that the regions of the neck and shoulder were chosen by 61% each, the lumbar spine by 56%, and the thoracic spine by 44% of the respondents; hence, these are the most prevalent MSDs. More than 2/3 of the respondents sat for more than 8 h on a daily basis, which increases the risk of the development of several disorders caused by inactivity and a sedentary lifestyle. The willingness of the respondents to participate in a health check-up and a workplace training program was extremely high since all of them wanted to attend the physical examinations and more than 90% of them would have liked to participate in a workplace training program in the future (Table 3).

### 3.2. SpinalMouse

A total of 76 subjects were eligible for the postIPE, 45 male and 31 female participants with a mean age of 32.78 ± 6.59 years. The frontal plane examinations of the spine in the sitting position presented small changes in the positions and ROMs of the segments and the complete spine. Only the left lateral-flexion (before median −0.85° (IQR: −2.65–−1.55°)–after median −2.15° (IQR: −4.15–0.1°)) and upright-to-left ROM (before median −1.4° (IQR: −3.7–1.3°)–after median −2.55° (IQR: −4.35–−0.11°)) showed a significant increase in degrees, where the values present an increase in the tilt of the pelvis on the right side. Although unfavorable changes can be seen in the lumbar region (in the LE, U and U–L) and the thoracic spine (in the U, RI, U–L and U–R), the complete inclination of the whole spine showed a non-significant increase in all the examined aspects—except in U—and all the segments showed a non-significant increase in the ROM of L–R. The upright (U) inclination of the spine decreased favorably (before median 1.45° (IQR: 0.5–2.35°)–after median 1.35° (IQR: 0.45–2.4°)), which indicates a more neutral position; however, this result was not significant (Table 4).

The frontal plane examinations of the spine in the standing position presented a few more significant changes in the position and ROM of the spine and its segments compared to the results measured in the sitting position. The favorable increase in the inclination of the spine was significant (*p* < 0.05) in all the examined aspects except in U, where the increase in degrees represents a small right inclination compared to the neutral position (before median 2.3° (IQR: 1.15–3.3°)–after median 2.4° (IQR: 1.3–3.7°)); however, this negative change was not significant. The active lateral-flexion ROM of the lumbar region also showed a significant increase in U–L (before median 20.95° (IQR: 15.25–25.9°)–after median 22.3° (IQR: 18.7–25.15°)) and L–R (before median −44.9° (IQR: −52.85–−36.95°)–after median −48.25° (IQR: −54.9–−42.9°)) as well as the total inclination of the spine (Table 5).

More remarkable results can be observed in relation to the measurement performed in the sagittal plane. The values measured in the upright (U) sitting position present a positive and significant impact on the posture since the thoracic kyphosis decreased (before median 27° (IQR 20.5–36.5°)–(after median 25° (IQR 18–33°)), the lumbar lordosis increased (before median −6.5° (IQR −14.5–0.5°)–after median −13° (IQR −18–−6°)), the anterior tilt of the pelvis was larger (before median 1° (IQR −4–8°)–after median 5° (IQR 1–14°)) and the anterior inclination of the spine (before median 2.5° (IQR 0–6°)–after median 1° (IQR 0–4°)) was decreased; thus, the posture was closer to the desired neutral position. Significant positive changes can be observed in flexion (F)—except for the position of the thoracic spine, where the overall inclination showed the largest increase in degrees (before median 49° (IQR 40–60°)–after median 65.5° (IQR 51.5–72°)). A small and non-significant increase in the extension (E) of the sacrum and hip, lumbar region and whole spine was observed; moreover, a significant decrease in the kyphosis of thoracic spine (before median 17.5° (IQR 11–26°)–after median 13° (IQR 5.5–20°)) was found. The extension was performed with an increased anterior tilt of the pelvis, larger lumbar lordosis and smaller thoracic kyphosis, which resulted in an increased total inclination of the spine in E. A significant increase in the estimated ROM in U–F was observed in all the regions (*p* < 0.001), except for the thoracic spine, which remained unchanged. Some interesting results for the estimated extension ROM in U–E were observed since an increased posterior pelvic tilt and decreased thoracic kyphosis can be seen with a decreased lumbar lordosis and decreased total inclination after the intervention. Based on a review of the measured values, we can conclude that the post-intervention sitting extension was performed with a lesser amount of anterior tilt of the pelvis; therefore, the core muscles were forced and able to manage larger loads. A smaller anterior tilt of the pelvis shifts the center of gravity more dorsally compared to the line of gravity and increases the resistance arm acting on the trunk; hence, it limits or decreases the available lumbar and thoracic extension. The estimated ROM from total extension to flexion (E–F) showed a statistically significant increase (*p* < 0.05) in all the examined regions in the sitting position (Table 6).

The most significant changes were observed in standing position in the sagittal plane. The distribution of the values measured in U in the sacrum–hip complex showed an increased anterior tilt of the pelvis, decreased thoracic kyphosis and increased lumbar lordosis (*p* < 0.05). The total inclination of the spine showed a more posterior position compared to the vertical axis before and, even more, after the intervention (before median −2° (IQR −3–0°)–after median −2° (IQR −4–−1°)). An increase in the angles measured in F can be seen in all the regions, and this increase was significant in the case of the sacrum/hip and total inclination (*p* < 0.001). The standing extension was performed with a decreased non-significant posterior pelvic tilt and a significant decrease in thoracic kyphosis (*p* < 0.05), and increases in lumbar lordosis (*p* < 0.001) and total inclination (*p* < 0.05) were observed. The estimated ROM in flexion (U–F) was significantly larger in the thoracic and lumbar spine (*p* < 0.05) as well as in the sacrum–hip and total inclination (*p* < 0.001). The estimated extension ROM of the sacrum–hip and lumbar regions decreased, although the ROM of the thoracic region and total inclination was increased; nonetheless, these changes were non-significant. The increase in estimated total ROM from extension to flexion (E–F) was significant in all the examined regions; therefore, it can be concluded that the mobility of the spine was increased as well as the flexibility of muscles. A stronger significance (*p* < 0.001) was observed for the mobility of the sacrum–hip complex (before median 34.5° (IQR 25–44.5°)–after median 40.5° (IQR 30.5–55°)) and the total inclination (before median 102° (IQR 92.5–108.5°)–after median 111.5° (IQR 101–123.5°)), possibly due to increased flexibility of the ischiocrural and paravertebral muscles (Table 7).

### 3.3. Physical Examination

The improvement of the expansion of the chest was significant at the levels of the axilla, the xyphoid process and the ribs (*p* < 0.001), which may be related to the improvement of spinal mobility. The results related to the Sit and Reach Test showed a significant improvement (*p* < 0.05), and, in the case of the second trial, the change was strongly significant (*p* < 0.001). The Y-Balance Test was primarily used to assess balance and coordination and, moreover, to predict proprioception, lower limb strength and core stability. Although the improvement of the reach distance for the right posteromedial direction was non-significant, all the other directions on both sides showed a significant change (*p* < 0.05), and among those, the reach distance for the left anterior direction showed the strongest significance (*p* < 0.001). The improvement of the static-isometric strength of the core muscles showed an impressive improvement in the Prone Plank, Left- and Right-Side Plank and Biering-Sorensen Test, where the level of significance was *p* < 0.001. The dynamic strength of the abdominal flexor muscles assessed using the Timed Abdominal Curl Test also presented a significant improvement in both time and repetition number, with *p* < 0.01. The analysis of the data measured during the physical examinations showed outstandingly good results; hence, it can be concluded that the intervention was extremely effective for the physical capabilities of the participants (Table 8).

Correlation analysis among variables related to the physical examination was performed. Changes in the inclination of the spine measured in sitting and standing positions both in the frontal and sagittal planes were analyzed in relation to the changes in the Sit and Reach Test and in Chest Expansion on three levels of measurement. Only one significant but negative correlation between the sitting frontal plane L-R inclination of the spine and CEA (rho = −0.303; *p* = 0.010) was found. A statistical analysis was also performed using the results related to Chest Expansion relative to the results related to the Plank, Side Plank, Timed Abdominal Curl and Modified Biering-Sorensen Tests. Among the analyzed variables, only one significant positive correlation was found between the CEA and Side Plank on the right side (rho = 0.299; *p* = 0.011). The most remarkable results were yielded by the correlation analysis of the balance and isometric muscle strength tests. Positive significant correlations were found between the differences measured in the Plank Test and YBT for the right posterolateral (rho = 0.327; *p* = 0.008) and left posterolateral (rho = 0.260; *p* = 0.032) directions; in the Left-Side Plank Test and YBT for the right anterior (rho = 0.364; *p* = 0.002) and right posterolateral (rho = 0.391; *p* = 0.001) directions; and in the Right-Side Plank Test and YBT for the right anterior (rho = 0.362; *p* = 0.002), right posterolateral (rho = 0.373; *p* = 0.002), and right posteromedial (rho = 0.247; *p* = 0.049) directions.

### 3.4. Post-Intervention Questionnaire

The postIQ was completed by 91 respondents (mean age, 33.52 ± 6.41 years; 51 male; 40 female) out of those who participated in the TP. The questions were intended to assess the subjective opinions of the participants about the effects of the TP. More than three quarters of the participants perceived their health as “Good” and fewer than a quarter of them as “Satisfactory” after the intervention. Although more subjects considered their condition to be better in terms of perceived health, a controversial result can be seen in terms of health behavior, since two-thirds of the respondents answered that they could do a great deal for their health and one-third of them responded that they could do little for their health. More than half of the respondents complained about musculoskeletal pain during the TP, and more than 80% of them indicated that these symptoms were decreased or eliminated. New symptoms appeared in two subjects, and only one person noticed that the symptoms had increased. The adherence to the recommended number of completed training sessions was not satisfactory since only 19.78% of the respondents claimed to have taken part in three training sessions weekly, and 70.33% of them marked “2 sessions weekly”. Despite the fact that the participants were not able to carry out the recommended 60 sessions, the TP proved to be effective according to the results of the physical examinations, whose benchmark was a minimum of 50% of the recommended sessions. The reasons for quitting or occasional participation were a lack of time (21.88%) and illness or delegacy (46.88%) among those 31 subjects who chose to respond to this question. A Likert scale was used to assess the satisfaction rate for the usefulness of the content of the TP, where 87.91% of the respondents answered “Very satisfied”, 10.99% answered “Satisfied” and 1.10% answered “Neutral”. The stretching and myofascial release techniques were incorporated in the daily routines of 93.41%, and the learned exercises were practiced at home on a regular basis by 84.62% of the respondents. The most popular exercises that were practiced at home were stretching (54.95%), SMR Trigger-Ball (52.75%), SMR Foam-Roller (51.65%) and exercises without tools (42.86%). Feedback on the TP revealed that the Net Promoter Score was excellent since none of the respondents were in the group of detractors, 5.5% of them were in the group of passives and 94.51% of them belonged to the group of promoters. Improved posture was perceived by 90%, improved physical endurance was perceived by 98.89% of the respondents, and 96.7% of them indicated that the New Spine program had helped to expand their knowledge in preventing problems caused by a sedentary lifestyle (Table 9). Based on the results of the physical examinations and the subjective feedback of the participants, the Training Program was effective in several aspects, including producing an increase in the level of daily physical activity, a reduction in musculoskeletal symptoms, and an improvement of posture and physical endurance, as well as muscle strength and flexibility, spinal mobility, balance and coordination.

## 4. Discussion

Our study aimed to analyze the effects of a Multimodal Workplace Training Program specifically designed for sedentary employees to reduce the risk of MSDs and LBP and, moreover, to decrease symptoms relating to these disorders in the case of involvement. Since the acute presence of complaints and symptoms was not a criterion for participation, the measurement of the level of pain using the Visual Analogue Scale (VAS) and the measurement of the level of functional disabilities using the Oswestry Disability Questionnaire (ODI) were disregarded. Despite an abundance of studies related to workplace health promotion and disease prevention in the literature, the comparison of these results is difficult due to the heterogeneity of the structures of these programs, of the target population, and of the applied examinations and training methods. None of the relevant studies contain such a long and complex program with similar examination methods and tools; therefore, the results related to the variables used in our study are difficult to compare.

According to the results of the systematic review of Sadler et al. [41], a reduction in spinal lateral-flexion ROM, decreased lumbar lordosis, and limited hamstring flexibility are significantly associated with the development of LBP; however, caution is advised in the interpretation of these results due to the mixed populations involved in the analyzed studies and the close proximity of the values to the upper borders of the confidence intervals. Although the authors also concluded that the association of the lumbar flexion and extension ROM, fingertip to floor distance, trunk flexor and extensor muscle strength and endurance with LBP was non-significant, definitive conclusions cannot be drawn due to the meta-analyses lacking power and generalizability caused by the low number of included studies [41]. Assuming that the aforementioned musculoskeletal factors are indeed risk factors for LBP, our program proved to be effective for the prevention of LBP, since changes in these properties were examined and significant positive changes were achieved in our study.

Jakobsen et al. [42] compared the effectiveness of 10-week workplace and home-based training on musculoskeletal pain, back extensor muscle strength and the self-rated use of analgesics among 200 female health-care workers. In addition to ergonomic training and education, high-intensity strength training was performed at the workplace under the supervision of an instructor, while home-based training was performed at home during leisure time using posters containing illustrations of the exercises. Despite the fact that the intervention and the training sessions—5 × 10 min a week—were shorter compared to our Training Program, both forms of training resulted in significant positive changes in outcome measures; however, between-group analysis revealed that the workplace-based intervention resulted in a significantly larger impact on the measured variables (*p* < 0.05) as well as on subjective parameters and adherence to the program (45%; 2.2 ± 1.1 sessions weekly). Since the strength of the back extensor muscles can be improved by relatively short interventions and short training sessions, we do believe that a longer program obviously results in a greater and more lasting impact. In accordance with the authors’ related results (80%) of those participants who reported musculoskeletal pain during our TP, 82.35% reported a reduction or elimination of musculoskeletal symptoms; therefore, this can be considered a similarly favorable result [42]. Based on our research experience, we believe that workplace interventions can indeed be more effective than individual or unsupervised home-based training since the supervision is professional, there is a scientifically sound schedule and progression, there are more motivation and group dynamics, there is a team-building effect, and even those who otherwise cannot be activated—since they may not be able or want to work out outside working hours—can be persuaded to do regular physical activity.

Similar exercises were used by Sipaviciene and Kliziene during a 20-week training program among patients affected by chronic LBP. The participants were randomly allocated to lumbar stabilization (*n* = 35) or lumbar muscle strengthening (*n* = 35) exercise program groups and performed the training twice a week for 45 min. The exercises and the length of the interventions were similar to those used in our program. Their results showed that both forms of training were effective in the reduction of functional disability, and in the improvement of the size of the cross-sectional area of the lumbar multifidus (CSA) and maximal isokinetic trunk flexor and extensor strength; moreover, these variables significantly correlated with LBP. However, both forms of training were effective; segmental stabilization proved to be superior to general strengthening since its effects lasted up to 12 weeks. These observations support the importance of the application of lumbar segmental stabilization, as well as its significant effect on core muscle strength in accordance with our results [16]. Based on the data found in the scientific literature, we consider it important to modify the examination protocol for our future studies. The division of subjects into acute, chronic and control subgroups may improve the comprehensibility of the impacts of such an intervention using pain-related questionnaires and scales, e.g., ODI and VAS. In addition, changes measured in the cross section of the core muscles (CSA) are also important measures; therefore, in our opinion, similar examination methods need to be applied in the future.

Based on the findings of Shariat et al. [43], stretching exercise training among three different interventions caused the most impressive positive outcome on MSD scores according to within-group analysis and compared to the control group at the end of the study. Their interventions were conducted among 147 white-collar workers and lasted for 6 months; sessions were performed once a day, three times a week, for as long as 10–15 min. The ergonomic modification training and the combined training (stretching and ergonomic modification) also showed significant improvement at the fourth-month follow-up examination. Since our training contained all these methods, we conclude that the application of ergonomic education as well as stretching exercises should be an essential part of a complex, multimodal program. Unfortunately, physical measures of flexibility were not measured by those authors; therefore, we can only assume that the effects of the stretching exercises contributed to the reduction of pain in our research, where they resulted in a significant improvement in spinal mobility and flexibility. In terms of pain reduction, we can only rely on participants’ positive feedback [43].

Functional resistance training was used by Cortell-Tormo et al. [44] in a randomized controlled trial among 24 female subjects affected by non-specific LBP to evaluate its effects on pain (VAS), disability (ODI), health-related quality of life (Short Form 36 Health Survey—SF-36) and physical fitness. The authors also applied exercises similar to ours (e.g., segmental stabilization, lunges and squats) in a progressive manner, as well as similar physical examination methods (e.g., static trunk extensor endurance, side plank, and timed abdominal curl tests) being used. Although the number of subjects was smaller and their intervention was shorter (12 weeks; two sessions weekly for 45–60 min) than ours, significant improvements were observed in the target group in all the measured variables compared to the control group except in the results related to the SF-36 [44]. Despite the methodological differences in the respective pieces of research, it can be concluded that functional exercises are a fundamental part of both preventive and therapeutic interventions in LBP or other musculoskeletal diseases. Based on our experience, it is important to highlight the proper and efficacious progression of exercises in order to prevent disadvantageous outcomes, overloading or further injuries. Notwithstanding the non-significant changes related to the SF-36, we believe that this questionnaire is important to use in our future workplace intervention, as our present subjects’ feedback contained overwhelmingly positive comments on changes in quality of life. It is noteworthy to mention that the subjects were asked to write their individual and subjective feedback on their experience related to pain, level of activity, posture and performance in the postIQ, and 43 out of 91 respondents (47%) indicated that their posture, endurance, wellbeing, productivity, quality of life and/or level of activity had improved. Moreover, they were also asked to write their comments on how the New Spine Program could be improved. Despite a lack of examples of possible answers, 47 respondents (51.64%) indicated that they would like to continue the TP instead of indicating any concrete changes.

In addition to the planned future changes in our research methodology mentioned above, we consider it important to restructure our program in line with the requests of both the employer and employee to make our TP continuously available to the entire staff and to increase the impact of the intervention. In order to create a workplace training program that covers all the training methods, aims and features that were used in this present study, several different types of training, sessions according to differentiated ability levels, professional human resources, adequate infrastructure and equipment, and the support of stakeholders are required.

According to our experience, any health-promotion project implemented in a workplace setting may require methodological changes since the researcher should follow the regulations of the employer; moreover, these studies should be performed in realistic instead of theoretically regulated circumstances. Although possible interference in the results that could have been caused by a random order of the tests was resolved in our study by the appropriate definition of the time required for complete recovery, the assessment of possible interferences in relation to different variations of test protocols could be an important and interesting topic for future studies.

Several statistical analyses were performed in order to determine the correlation of different variables measured in our study. Hamstring flexibility and spinal mobility are one pair of those variables that are in the focus of experts studying LBP. Although a systematic review of Sadler et al. [41] concluded that the restriction of the lateral flexion of the spine and hamstring flexibility are predictors of LBP, our findings and those of previous studies showed a weak or non-significant relationship between these variables; therefore, we suggest that further RCTs should be performed among symptomatic and asymptomatic LBP patients [45,46]. Another interesting aspect of the effects of LBP is the alteration of the breathing mechanism. Since the diaphragm muscle plays an important role in lumbar stabilization, the pain-reactive inhibition of the core musculature may cause a limitation of chest expansion and respiratory function. Previous studies have shed light on the relationship between chest expansion and core muscle strength as well as the importance and effectivity of respiratory training in LBP [47,48]. Although the chest expansion measured on each level and all the trunk muscle strength tests showed a statistically significant improvement, a significant correlation was only found between the differences measured in the Right-Side Plank Test and Chest Expansion on Axillar level. Our findings need to be considered with precaution due to the lack of direct examination of variables related to pulmonary function, since, in our opinion, that would provide a better characterization of the function of the diaphragm instead of the intercostal and other synergist muscles. In contrast to the associations presented above, we found a remarkable correlation between the changes in isometric core strength and changes in balance measured by the Y-balance Test. According to the findings of Hooper et al. [49], the balancing ability is reduced in LBP; this can be easily examined using the YBT. No correlation between YBT scores and pain intensity, fear avoidance or disability scores was found in their study, but the correlation with core strength was not analyzed. We believe that the correlation found in our study can be explained by the patho-mechanism of LBP, since the pain-reactive inhibition and weakening of the multifidus muscles may result in poor proprioception; therefore, the reduction of motor control deteriorates balancing strategies. Furthermore, weakness, atrophy and inhibition of the stabilizers of the lumbar spine may trigger further pain and instability of the intervertebral joints. Based on these patho-anatomic facts and the association found between balance and core strength, we recommend the application of segmental stabilization exercises as well as proprioceptive training for the prevention or treatment of LBP.

It is important to mention that the prevention of MSDs and LBP has to be started as soon as the population starts facing the risk factors for these disorders; hence, all interventions created for adults could be more effective if the preventive work was started in childhood or adolescence. The systematic review conducted by Miñana-Signes et al. [50] highlighted the lack of research and evidence for best practices among school-aged children; however, the prevention of the development of MSDs could be highly effective at this stage of life. Similarly to in workplace settings, a large number of the population at risk can be targeted in school settings. Moreover, childhood and the teenage stages of life are more suitable in which to receive, process and learn information and practices related to health and a health-conscious lifestyle [50]. We agree with the authors that early multidimensional and multidisciplinary childhood prevention may reduce the number of adults affected by these disorders and may improve the outcomes of preventive strategies implemented among adults; therefore, the application of a new version of our TP designed for children is also among our future plans.

### Limitations

The limitations of this study were the lack of sufficient time and a proper venue for the physical examinations and training; the low level of adherence caused by a hectic workload; and the relatively large drop-out rate caused by unplanned workloads, sick leave and official delegations. Problems related to sufficient time and an adequate venue could be managed by the willingness, support and efforts of the management of the company. The implementation of a restructured continuous TP may increase adherence and decrease the drop-out rate, since participants may decide to switch between training types and difficulty levels at any time.

It is important to highlight the confounding factors caused by the strict regulations of the employer observed during the development of the study protocol. The timetable and the order of the tests were created by the researchers, and the co-workers delegated by the company according to the specific regulations of the employer. Due to the limited time, space and human resources allowed and available for the physical examination, we were compelled to use a random order for the tests in order to involve a large number of employees during their working hours using a relatively short period of time. Researchers should carefully consider the effect factors caused by the requirements of the employer during the process of the construction and design of the study and should manage any possible risk of research bias.

## 5. Conclusions

Based on our results and those of previous studies, segmental stabilization, stretching, self-myofascial release and functional training have a significant positive effect on pain, on disability, on posture, on muscle strength and on balance; therefore, these can be effective methods for the prevention and treatment of musculoskeletal diseases and low back pain. The combination of these techniques probably enhances these effects; hence, Multimodal Progressive Functional–Proprioceptive Training can be recommended among individuals affected by these problems; moreover, as an advantageous intervention, it can be effectively used among sedentary employees in workplace settings. Further randomized controlled trials are recommended, to gain a deeper understanding of the effects of such an intervention.

## Figures and Tables

**Table 1:** Inclusion and exclusion criteria.

Inclusion Criteria	Exclusion Criteria
white-collar (sedentary) worker of the company	lack of completion of pre-intervention questionnaire
presence of acute or chronic non-specific LBP	lack of completion of pre-intervention physical examination
previous LBP episode during the last twelve months	the number of training sessions completed by the participant < 30 sessions
having no other known, diagnosed spinal, internal organ or other musculoskeletal disorders	lack of completion of post-intervention physical examination
age between 20 and 60 years	lack of signature for informed consent
lack of specific LBP	
having willingness and/or ability to participate in the Training Program	

**Table 2:** Structure of the Training Program.

Week	Phases	Recommended Number of Sessions
1–2.	Ergonomic instructions, stretching techniques and self-myofascial release	6
3–4.	Segmental stabilization of lumbar spine	6
5.	Static strengthening of core musculature	3
6.	Segmental stabilization combined with dynamic exercises	3
7–8.	Basic functional exercises in different positions	6
9–20.	Functional training	36

**Table 3:** The distribution of participants by percentage among specific answer categories for multiple-choice questions in preIQ (*n* = 236).

Question/Answer	Percent (%)
Looking back at the last 12 months, what do you think your health is like in general?	
Good	65.54
Satisfactory	31.91
Bad	2.55
How much do you think you can do for your health?	
I can do a lot	99.14
I can do a little	0.86
If you have had any musculoskeletal complaints (low back pain, neck pain, back pain, etc.) during the last 12 months please indicate the location of the pain?	
Neck	61.01
Thoracic spine	44.06
Lumbar spine	55.93
Shoulder	61.01
Elbow	11.86
Wrist and hand	11.08
Hip	13.55
Knee	31.77
Ankle and foot	13.55
How much time do you spend sitting on an average day? This includes time when you sit at your desk during your work hours or visit your friends and sit with them, maybe study, watch TV, or eat?	
Less than 1 h	0.42
More than 2 h but not more than 5 h	4.68
More than 5 h but not more than 8 h	26.80
More than 8 h	68.08
Would you like to take part in a workplace health check-up?	
Yes	100.00
No	-
Would you like to participate in a training program led by physiotherapists in your workplace?	
Yes	92.34
No	7.65

**Table 4:** Medians and interquartile ranges of total inclination and lumbar, thoracic and sacrum/hip inclinations in degrees measured before and after the intervention in sitting position in the frontal plane: left lateral-flexion (LE), upright (U), right lateral-flexion (RI) and estimated ROM from upright to flexion (U–F), upright to the left (U–E), upright to the right (U–R) and left to the right lateral-flexion (L–R) (*n* = 76).

Frontal Sitting								
Position	Segment	Before			After			*p*-Value
		Q1	Median	Q3	Q1	Median	Q3	
LE	Sac/Hip	−2.65	−0.85	1.55	−4.15	−2.15	0.10	0.012 *
	Thoracic	19.80	24.40	27.55	21.45	24.90	29.75	0.243
	Lumbar	10.45	15.75	18.45	10.40	14.65	17.65	0.107
	Inclination	15.75	19.05	22.20	16.75	19.35	23.65	0.465
U	Sac/Hip	−1.20	0.10	1.55	−1.20	0.05	1.30	0.783
	Thoracic	−3.30	−1.55	0.45	−3.70	−1.80	0.30	0.648
	Lumbar	0.05	2.50	4.65	0.60	2.55	4.15	0.827
	Inclination	0.50	1.45	2.35	0.45	1.35	2.40	0.665
RI	Sac/Hip	−1.95	−0.50	1.85	−2.20	−0.55	2.00	0.791
	Thoracic	−30.85	−27.20	−22.15	−32.3	−26.85	−21.05	0.686
	Lumbar	−17.45	−13.05	−9.45	−16.4	−13.85	−10.30	0.590
	Inclination	−19.75	−16.9	−13.95	−20.4	−17.7	−15.35	0.387
U–L	Sac/Hip	−3.70	−1.40	1.30	−4.35	−2.55	−0.15	0.006 *
	Thoracic	20.50	26.55	30.20	22.35	26.35	31.25	0.282
	Lumbar	8.25	12.45	17.55	8.55	12.05	15.20	0.161
	Inclination	−21.70	−17.30	−13.95	−22.30	−18.10	−15.30	0.296
U–R	Sac/Hip	−2.50	−0.80	1.50	−2.10	−0.45	1.65	0.917
	Thoracic	−30.60	−25.90	−21.40	−30.45	−25.20	−20.55	0.860
	Lumbar	−19.70	−15.55	−11.05	−19.30	−16.45	−12.30	0.771
	Inclination	15.30	19.15	21.35	16.80	19.50	21.80	0.514
L-R	Sac/Hip	−2.20	0.30	3.40	−0.30	1.75	4.65	0.098
	Thoracic	−58.45	−50.70	−44.05	−60.65	−51.50	−44.90	0.934
	Lumbar	−34.05	−27.50	−21.95	−33.35	−27.65	−23.15	0.576
	Inclination	30.10	35.60	41.80	32.50	37.60	44.45	0.370

* Significant results (*p* < 0.05); negative values indicate right direction angles, while positive values denote left direction angles of the spine.

**Table 5:** Medians and interquartile ranges of total inclination and lumbar, thoracic and sacrum/hip inclinations in degrees measured before and after the intervention in standing position in the frontal plane: left lateral-flexion (LE), upright (U), right lateral-flexion (RI), estimated ROM from upright to flexion (U–F), upright to the left (U–E), upright to the right (U–R) and left to the right lateral-flexion (L–R) (*n* = 76).

Frontal Standing								
Position	Segment	Before			After			*p*-Value
		Q1	Median	Q3	Q1	Median	Q3	
LE	Sac/Hip	−6.65	−4.05	−1.90	−7.05	−4.50	−2.15	0.925
	Thoracic	19.00	23.85	28.35	22.00	26.75	30.95	0.003 *
	Lumbar	19.30	23.25	29.20	21.60	26.25	30.10	0.058
	Inclination	25.30	29.45	35.50	27.95	32.60	36.65	0.002 *
U	Sac/Hip	−1.45	0.65	2.25	−1.80	0	2.25	0.385
	Thoracic	−4.00	−1.15	1.65	−2.60	−0.45	2.25	0.251
	Lumbar	0.55	3.05	6.60	0.60	2.65	5.30	0.541
	Inclination	1.15	2.30	3.30	1.30	2.40	3.70	0.124
RI	Sac/Hip	0.25	2.85	4.70	0.25	2.05	5.30	0.432
	Thoracic	−31.05	−26.55	−21.45	−31.50	−25.25	−19.05	0.844
	Lumbar	−25.40	−21.70	−18.20	−27.30	−24.00	−19.85	0.078
	Inclination	−31.90	−26.25	−22.30	−33.10	−28.80	−24.85	0.023 *
U–L	Sac/Hip	−7.15	−4.85	−2.40	−7.40	−4.70	−1.85	0.522
	Thoracic	19.10	24.45	30.50	22.85	26.95	31.15	0.057
	Lumbar	15.25	20.95	25.90	18.70	22.30	25.15	0.044 *
	Inclination	−32.20	−28.45	−23.55	−33.95	−29.30	−26.75	0.009 *
U–R	Sac/Hip	−0.75	1.35	3.80	−0.55	2.20	5.00	0.579
	Thoracic	−28.95	−24.45	−20.90	−30.95	−25.00	−18.20	0.560
	Lumbar	−29.55	−25.40	−21.00	−31.35	−25.70	−22.70	0.225
	Inclination	24.00	28.95	33.45	26.95	31.65	35.25	0.017 *
L–R	Sac/Hip	4.05	6.50	9.90	4.40	7.70	10.65	0.671
	Thoracic	−57.10	−50.60	−41.05	−61.30	−51.95	−42.95	0.282
	Lumbar	−52.85	−44.90	−36.95	−54.90	−48.25	−42.90	0.012 *
	Inclination	48.00	56.15	65.35	53.60	62.50	67.80	0.006 *

* Significant results (*p* < 0.05); negative values indicate right-direction angles, while positive values denote left-direction angles of the spine.

**Table 6:** Medians and interquartile ranges of total inclination and lumbar, thoracic and sacrum/hip inclinations in degrees measured before and after the intervention in sitting position in the sagittal plane: upright (U), flexion (F), extension (E), and estimated ROM from upright to flexion (U–F), upright to extension (U–E) and extension to flexion (E–F) (*n* = 76).

		Sagittal Sitting						
Position	Segment	Before			After			*p*-Value
		Q1	Median	Q3	Q1	Median	Q3	
U	Sac/Hip	−4.0	1.0	8.0	1.0	5.0	14.0	<0.001 *
	Thoracic	20.5	27.0	36.5	18.0	25.0	33.0	0.006 *
	Lumbar	−14.5	−6.5	0.5	−18.0	−13.0	−6.0	<0.001 *
	Inclination	0	2.5	6.0	0	1.0	4.0	0.001 *
F	Sac/Hip	4.5	11.5	21.0	12.0	26.5	34.0	<0.001 *
	Thoracic	61.0	67.5	74.0	58.5	65.5	72.5	0.593
	Lumbar	19.0	25.5	29.00	22.0	27.0	31.0	<0.001 *
	Inclination	40.0	49.0	60.	51.5	65.5	72.0	<0.001 *
E	Sac/Hip	−2.5	3.0	7.0	−1.0	5.0	11.0	0.038 *
	Thoracic	11.0	17.5	26.0	5.5	13.0	20.0	<0.001 *
	Lumbar	−41.0	−34.0	−27.5	−42.5	−35.0	−28.0	0.103
	Inclination	−26.0	−22.0	−18.0	−26.0	−23.0	−19.0	0.659
U-F	Sac/Hip	1.0	8.5	19.0	6.0	19.0	28.0	<0.001 *
	Thoracic	27.5	40.0	46.0	33.0	40.0	48.0	0.082
	Lumbar	25.0	31.5	38.5	33.0	38.0	45.0	<0.001 *
	Inclination	38.0	45.5	55.5	48.5	63.0	71.5	<0.001 *
U-E	Sac/Hip	−6.5	1.0	6.0	−6.5	−2.0	6.0	0.098
	Thoracic	−14.5	−10.0	−5.0	−18.5	−11.0	−6.5	0.158
	Lumbar	−35.5	−25.0	−19.0	−27.5	−22.5	−16.0	0.003 *
	Inclination	−29.0	−25.0	−21.0	−28.0	−24.0	−20.0	0.207
E-F	Sac/Hip	1.5	8.0	19.5	6.0	19.5	29.5	<0.001 *
	Thoracic	38.0	48.0	56.0	46.0	53.0	60.0	0.004 *
	Lumbar	51.5	59.5	65.5	54.0	63.5	69.0	0.001 *
	Inclination	60.0	73.0	81.0	73.0	86.0	98.0	<0.001 *

* Significant results (*p* < 0.05); negative values indicate lordotic angles, while positive values denote kyphotic angles of the spine.

**Table 7:** Medians and interquartile ranges of total inclination and lumbar, thoracic and sacrum/hip inclinations in degrees measured before and after the intervention in standing position in the sagittal plane: upright (U), flexion (F), extension (E), and estimated ROM from upright to flexion (U-F), upright to extension (U-E) and extension to flexion (E-F) (*n* = 76).

		Sagittal Standing						
Position	Segment	Before			After			*p*-Value
		Q1	Median	Q3	Q1	Median	Q3	
U	Sac/Hip	7.0	13.0	18.0	9.0	15.0	20.0	0.004 *
	Thoracic	29.0	36.0	46.0	25.0	36.0	40.5	0.003 *
	Lumbar	−33.0	−27.0	−21.5	−34.0	−29.0	−23.5	0.034 *
	Inclination	−3.0	−2.0	0	−4.0	−2.0	−1.0	0.001 *
F	Sac/Hip	25.0	29.0	40.0	33.5	42.0	49.5	<0.001 *
	Thoracic	56.0	62.0	69.0	55.5	63.0	69.0	0.518
	Lumbar	20.5	25.5	31.5	22.0	26.0	31.5	0.218
	Inclination	61.5	71.0	78.0	69.5	80.0	88.5	<0.001 *
E	Sac/Hip	−9.0	−3.5	3.5	−8.0	−1.5	5.5	0.086
	Thoracic	15.5	25.0	31.0	10.0	19.5	28.0	0.001 *
	Lumbar	−46.5	−40.0	−34.0	−49.0	−43.0	−36.5	<0.001 *
	Inclination	−37.0	−31.5	−26.0	−38.0	−32.5	−27.0	0.020 *
U–F	Sac/Hip	10.5	21.0	26.0	17.5	26.0	36.5	<0.001 *
	Thoracic	17.5	25.0	32.0	20.5	27.5	37.0	0.030 *
	Lumbar	48.0	52.0	60.5	50.5	55.0	62.0	0.012 *
	Inclination	63.5	73.5	78.5	72.0	81.0	90.0	<0.001 *
U–E	Sac/Hip	−22.0	−16.0	−8.0	−21.0	−15.5	−9.0	0.676
	Thoracic	−19.0	−11.5	−5.0	−23.0	−14.5	−6.5	0.367
	Lumbar	−16.0	−12.0	−7.5	−20.0	−11.0	−7.0	0.348
	Inclination	−35.0	−28.5	−24.0	−35.0	−29.5	−26.0	0.156
E–F	Sac/Hip	25.0	34.5	44.5	30.5	40.5	55.0	<0.001 *
	Thoracic	30.0	38.0	48.0	35.0	42.5	49.5	0.025 *
	Lumbar	59.0	65.5	74.0	62.0	68.5	77.5	0.001 *
	Inclination	93.5	102.0	108.5	101.0	111.5	123.5	<0.001 *

* Significant results (*p* < 0.05); negative values indicate lordotic angles, while positive values denote kyphotic angles of the spine.

**Table 8:** Medians and interquartile ranges (IQRs) of parameters measured before and after the intervention (*n* = 76).

Physical Examination	Before		After		*p*-Value
	Median	IQR	Median	IQR	
Chest expansion (CEA) (cm)	4.5	2.25	5.0	2.5	<0.001 *
Chest expansion (CEX) (cm)	5.0	2.0	7.0	2.5	<0.001 *
Chest expansion (CER) (cm)	5.5	2.0	6.0	2.3	<0.001 *
Sit and Reach 1st trial (cm)	16.8	15.5	20.0	18.5	0.010 *
Sit and Reach 2nd trial (cm)	17.5	17.5	20.5	19.0	<0.001 *
Sit and Reach average (cm)	17.1	17.3	19.3	19.0	0.001 *
YBT Right Anterior (cm)	58.0	9.5	62.0	7.0	0.004 *
YBT Right Posteromedial (cm)	87.0	14.0	88.0	11.0	0.061
YBT Right Posterolateral (cm)	91.0	14.5	95.0	14.0	0.019 *
YBT Left Anterior (cm)	57.0	10.0	61.0	10.0	<0.001 *
YBT Left Posteromedial (cm)	87.0	17.0	90.5	14.0	0.034 *
YBT Left Posterolateral (cm)	91.0	18.8	96.0	14.0	0.023 *
Prone Plank (s)	69.0	37.0	99.0	59.5	<0.001 *
Side Plank Left (s)	32.2	17.0	59.5	25.0	<0.001 *
Side Plank Right (s)	31.7	15.2	56.0	23.0	<0.001 *
Timed Abdominal Curl Test (rep)	29.0	24.0	34.5	27.5	0.001 *
Timed Abdominal Curl Test (s)	93.0	60.0	108.0	84.0	0.004 *
Modified Biering-Sorensen Test (s)	90.0	45.0	154.0	85.0	<0.001 *

* Significant results (*p* < 0.05).

**Table 9:** The distribution of participants by percentage among specific answer categories of simple- and multiple-choice questions in postIQ (*n* = 91).

Question/Answer	Percent (%)	*n*
Looking back at the last 12 months, what do you think your health is like in general?		91
Good	76.92	
Satisfactory	23.08	
How much do you think you can do for your health?		91
I can do a lot	67.03	
I can do a little	32.97	
Looking back at the last 20 weeks of the Training Program, did you have any musculoskeletal complaints (low back pain, neck pain, back pain, etc.)?		91
Yes	53.85	
No	46.15	
If you answered yes to the previous question, please choose from the following!		51
Symptoms decreased	72.55	
Symptoms eliminated	9.8	
Symptoms increased	1.96	
Symptoms remained	11.76	
New symptoms appeared	3.92	
How regularly did you participate in the training sessions?		91
3 sessions weekly	19.78	
2 sessions weekly	70.33	
1 session weekly	8.79	
I quitted	1.1	
If you rarely participated or quitted the Training Program, what was the reason?		32
Due to a major interruption (illness, leave, delegacy).	46.88	
I have no time	21.88	
Other	31.25	
How useful do you find what you learned during the Training Program? Please mark on a scale 1–5!		91
3	1.10	
4	10.99	
5	87.91	
Have you been able to incorporate the stretching exercises and myofascial-release techniques you learned during the Training Program into your daily routine?		91
Yes	93.41	
No	6.59	
Did you manage to practice the learned exercises at home on a regular basis?		91
Yes	84.62	
No	15.38	
If you answered yes to the previous question, which exercises are these? (multiple-choice)		91
Exercises without tools	42.86	
SMR Foam-roller	51.65	
SMR Trigger-ball	52.75	
Suspension Trainer	6.59	
Swiss ball and soft-ball	10.99	
Stretching	54.95	
How much would you recommend the New Spine Program to your colleagues? Please mark on a scale 0–10!		91
7	1.10	
8	4.40	
9	12.09	
10	82.42	
In your opinion, has your posture improved as a result of the Training Program?		90
Yes	90	
No	10	
In your opinion, has your physical endurance improved as a result of the Training Program?		90
Yes	98.89	
No	1.11	
In your opinion, has the New Spine program helped to expand your knowledge in preventing problems caused by a sedentary lifestyle?		91
Yes	96.7	
No	3.30	

## Data Availability

The data presented in this study are available on request from the corresponding author. The data are not publicly available due to further analysis of the data acquired with the support of the GINOP-2.3.2-15-2016-00005 project.
